# Managing Bony Defects of the Shoulder Joint that Occur in Association with Dislocation

**DOI:** 10.2174/1874325001711011245

**Published:** 2017-11-10

**Authors:** Jonathan Brian Yates, Muhammad Naghman Choudhry, Mohammad Waseem

**Affiliations:** Blackpool Teaching Hospitals NHS Foundation Trust Ringgold standard institution - Trauma and Orthopaedics Blackpool, Blackpool, United Kingdom of Great Britain and Northern Ireland, UK

**Keywords:** Hill-Sachs lesion, Shoulder dislocation, Glenoid defects, Shoulder stability, Glenoid track theory, Shoulder surgery

## Abstract

**Background:**

Defects to the articular surface of the humeral head have been known to be associated with shoulder dislocation since the 19^th^ century. It wasn't until 1934 that the first description of the ubiquitous compression fracture of the posterolateral humeral head that occurs with traumatic anterior instability appeared. From 1940, this defect became referred to as a Hill-Sachs lesion after the investigators who reported the condition. The significance of, and therefore treatment of, these and other such bony defects around the shoulder joint has been hotly debated.

**Methods:**

We reviewed the available current literature to determine and report on the most up to date concepts and treatment techniques being used to manage bony defects of the shoulder.

**Results:**

Numerous surgical options have been proposed to manage bony defects of the shoulder, including a variety of defect-filling procedures, with good outcomes. However, the small numbers and diversity of case mix makes for difficult comparisons.

**Conclusion:**

We are currently developing a greater appreciation of how both the humeral and glenoid defects interact and therefore should be assessed and addressed simultaneously in order to improve patient outcomes. More research and collaboration is needed to determine the optimal method of assessing and managing these patients.

## 1 INTRODUCTION

Defects within the humeral head have been known to be associated with glenohumeral dislocation since the 19^th^ century. It wasn't until 1934 that the first description of the ubiquitous “compression fracture of the posterolateral humeral head associated with traumatic anterior instability” appeared [1]. From 1940, this defect became referred to as a Hill-Sachs lesion (HSL) after the investigators who had first reported it [2]. Glenoid defects, when deemed appropriate, are usually addressed through the open Latarjet procedure, also known as the Bristow-Lartarjet or Bristow procedure [3]. Numerous options have been proposed for HSLs managed surgically, including a variety of defect-filling procedures. We are currently developing a greater appreciation of how both the humeral and glenoid lesions interact and therefore should be assessed and addressed simultaneously in order to improve patient outcomes. This review will look at the current theories adopted and novel treatments suggested for managing these lesions.

## 2 SIZE OF HILL-SACHS LESION

In order for any bony defect in the glenohumeral joint to become clinically symptomatic, it must first cause an abnormal interaction within the joint. The engagement of a HSL with the glenoid depends on its size and location relative to the glenoid. The average size of HSL is 22mm in width and 5mm in depth [4]. The critical dimensions of HSLs suggested to cause instability are those which are 4cm long by 0.5cm deep (a medium lesion) and 4cm long by 1.0cm deep (a large lesion), greater than 20-25% of the humeral head surface, deeper than 16% of the humeral head diameter and with a volume greater than 250mm^3^ or 1000mm^3^. [4] Biomechanical studies suggest that HSLs as small as 12.5-25% with concomitant glenoid bone loss can affect shoulder stability. They found that shoulders with large HSLs (37.5-50%) may benefit from allograft transplantation to restore shoulder stability [5]. A more horizontal orientation of HSL on CT scan has also been shown to predict the likelihood of engagement of the HSL [6].

## 3 EPIDEMIOLOGY

The incidence of HSLs appears to be directly proportional to the frequency of dislocation. Incidence has been observed to raise from 65-67% after a primary dislocation to 84-93% after a recurrent dislocation [7]. Its detection is reliant on the imaging modality used since small HSLs might be missed on plain radiographs, which may be seen on a magnetic resonance imaging (MRI) scan. The prevalence of symptomatic HSLs following unilateral recurrent anterior shoulder dislocations has been found to be around 7% [8].

## 4 PATHOANATOMY

The HSL occurs when the humeral head dislocates from the glenoid, engaging the relatively harder glenoid rim leaving an impression fracture on the humeral head. Two main factors which determine the severity of the resultant HSL are the frequency of episodes and the force generated upon dislocation. In addition, the position of the humerus at the time of dislocation is important with regard to location, depth, and orientation of the HSL. The capsule and labrum become increasingly attenuated with each episode contributing to recurrent instability and HSL. Dislocation can occasionally lead to injury of the subscapularis tendon or an anterior labroligamentous periosteal sleeve avulsion (ALPSA) lesion [2]. One must be mindful of the bipolar phenomenon, where there are defects formed in both the glenoid and humeral head as both must be addressed to achieve overall stability.

**Fig. (1):**
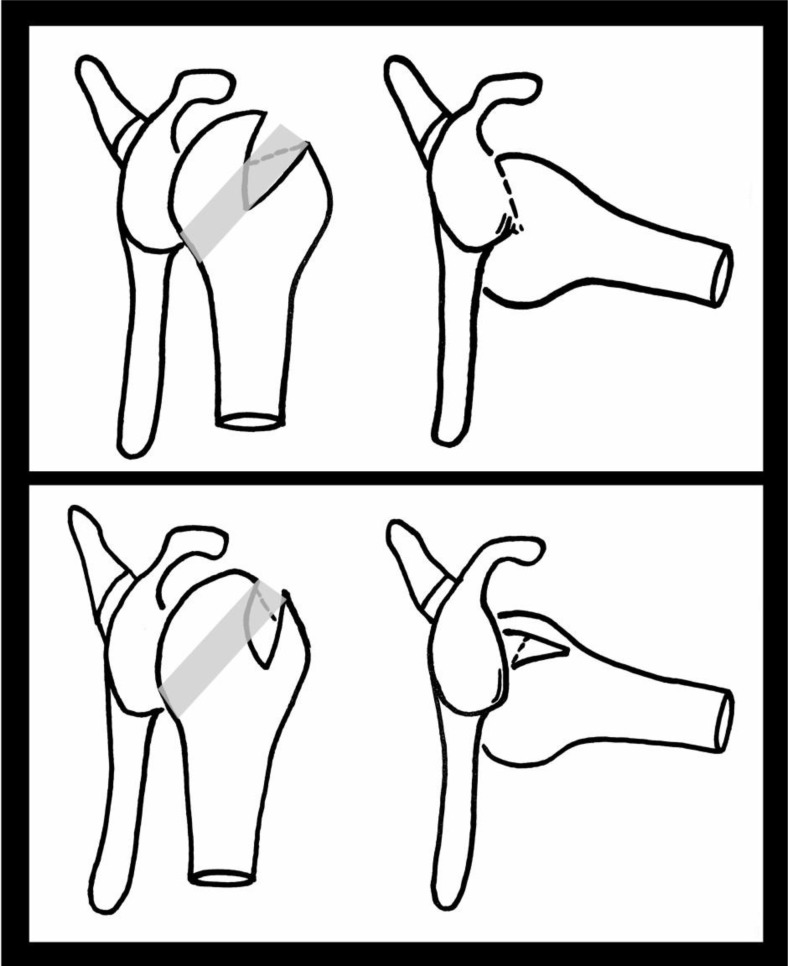
Glenoid track, engaging and none engaging HSLs. The upper and most left image shows the shoulder joint in neutral and then, on the right, in abduction and external rotation. The shaded area on the humerii shows the glenoid track. The lesion within the humeral head in the top image can be seen to engage the glenoid as it moves parallel to it as it is more medial than the glenoid track. The lower image demonstrates how this is much less likely to occur in a more vertical orientation and with the lesion sitting within the track *i.e.* a none-engaging HSL.

The concept of an articular arc deficit explains why some patients feel their shoulder is subluxing or dislocating when it is not clinically. The mismatch created by bony defects between the humeral head and glenoid alters their normal articular arc length [9]. HSLs that engage the anterior corner of the glenoid with the shoulder in 90° of abduction and external rotation are termed “engaging HSLs”. None-engaging lesions can cause symptoms in non-functional shoulder positions [10] (Fig. **1**).

## 5 GLENOID TRACK THEORY

With respect to HSLs, any coexisting glenoid defect will undoubtedly affect management and prognosis. The concept of the glenoid track is based on the orientation and location of lesions that lead to an engaging HSL [11]. The glenoid track is essentially the contact zone which occurs as the glenoid shifts along the posterior margin of the humeral head. (Fig. **1**) This occurs when the shoulder is in 60^°^ abduction and full external rotation and moves along the end range of motion. The glenoid track takes into account both glenoid and humeral defects. The track has been measured to be 84% the width of the glenoid, which is the distance from the contact area to the medial border of the rotator cuff footprint [12]. Therefore, if the measured width of HSL is larger than the glenoid track size then it is high risk for being an engaging lesion or “off-track” lesion. An off-track when compared to an on-track HSL does appear to be a significant risk factor for recurrence of symptoms and need for revision surgery after arthroscopic Bankart repair [13]. Biomechanical studies on cadavers support the glenoid track theory and also that a Bankart repair alone is unlikely to fully address the defect [14]. Clearly, treating lesions outside the glenoid track requires prevention of engagement to prevent instability. It has been suggested that this may be addressed at the glenoid side through a Latarjet procedure or on the humeral side through a rotational humeral osteotomy thereby moving the track position altogether [15]. The rotational humeral osteotomy whilst a well-established treatment for osteoarthritis can in itself cause osteoarthritis and so more work is needed to determine the exact risks of this in this situation. Understanding of and the utilisation of the glenoid track theory in both classification and in treating patients is currently under investigation.

## 6 CLINICAL ASSESSMENT

A thorough history and examination are needed to fully comprehend the characteristics of any bony shoulder instability and hence plan appropriate imaging and subsequent treatment.

## 7 HISTORY

Patients with instability will often complain of deep shoulder pain with mechanical symptoms such as crepitus, catching and clicking. The mechanism of injury and position of dislocation help delineate the type of dislocation and possible related soft tissue and bony injuries. Activities that place the shoulder in abduction, externally rotation and often with an extended arm and axial loading can lead to indirect anterior glenohumeral instability (*e.g.* in rugby players). Any high energy dislocation with recurrent episodes of instability associated with minimal force or in the mid-range of shoulder motion should be suspicious for bony deficiency [4]. With multiple dislocations, the history should focus on the force required to dislocate, the frequency of dislocations, method of relocation (self-reduction or attendance at a hospital) and the length of time from last dislocation. Dislocations that recur progressively easily on daily basis are likely to have large HSLs and combined glenoid defects [4]. Factors and activities that increase the chances of recurrence should be identified and reduced. Seizures, recurrent falls, alcoholism, drug dependency and activities that require arm abduction and external rotation are all known risk factors. The patient's occupation often dictates the goals of treatment in terms of functional range of motion for his or her occupation. Any previous surgical intervention should also be noted [4].

## 8 EXAMINATION

Clinical examination consists of checking the range of motion, power of deltoid and rotator cuff muscles and special instability tests. There may be reduced range of motion due to apprehension and there may be reports of pain secondary to arthritis or instability. Bony defects are more likely if a positive apprehension test occurs in mid-range. During the apprehension and relocation test one may also feel crepitus [16]. This grinding sensation may also be noted during the load and shift test (a modification of the shoulder drawer test), suggesting that the normal smoothness of the glenoid lip has been lost. There will be decreased resistance during translation showing a defect in glenoid cavity. This translation test also helps delineate laxity [17]. The presence of a sulcus sign suggests inferior instability. One must perform a thorough neurovascular examination and also rule out other injuries *e.g.* rotator cuff tear, bicep tendon pathology, superior labrum anterior-posterior (SLAP) lesions, axillary nerve injury or vascular injuries. In all cases, an examination under anesthesia precedes the actual surgery to confirm the clinical findings and imaging studies as well as to assess range of motion and document the pattern of instability without muscle guarding and apprehension.

## 9 IMAGING

Patients with shoulder instability should have at least three plain radiographs, including a true anterior-posterior, scapula lateral and axillary views. The Stryker notch and the internal rotation views are the most accurate radiographic views for diagnosing HSLs. The West Point view is useful in detecting glenoid rim lesions. However, these views may still not be sensitive enough to detect small defects [18]. One study looked at the inter-observer reliability in diagnosing osseous lesion in 10 patients with primary anterior shoulder dislocation. They compared plain radiographs with computed tomography (CT) scans [19]. The study concluded that radiographs are inferior to CT scans for assessing osseous lesions. The authors suggested performing a CT scan of the shoulder after primary dislocation to apply the correct treatment early potentially avoiding further dislocations [19]. Another study compared ultrasound (US) with CT arthrography (CTA) and that with arthroscopy in 92 patients. US showed 94% (81 of 86) accuracy when compared with CTA and an accuracy of 91% (79 of 86) in CTA when compared with arthroscopy. Ultrasound is therefore a valuable diagnostic tool in detecting HSLs, with several benefits including low cost and the ability to obtain dynamic, multiplanar images. Limitations include a dependence on operator experience and limited visualisation of intra-articular structures [20]. MRI (or MR arthrogram) has the advantage of detecting soft tissue pathology that may need to be addressed during surgical intervention and can certainly be a tool in approximating the amount of bone loss. In a double-blind, prospective study by Denti and colleagues, MRI showed accuracy of 87% when compared to arthroscopy. This is lower when compared to CTA as mentioned above [21]. A study looking into MRI evaluation of bone lesions on both the glenoid and the humerus showed an accuracy of 84.2% in predicting engagement of that lesion when compared to arthroscopy [22]. CT scan with digital subtraction remains the gold standard for the evaluation of a HSL as it allows the most accurate measures of location and size of lesions. However, Pagnani *et al* did show that the measurements made by CT are no better than those made during arthroscopy and there is also the tendency for CT to overestimate the size of larger glenoid defects [23].

## 10 CLASSIFICATION

Hill-Sachs lesions are classified into mild, moderate and severe groups *i.e.* mild, 2.0cm long and 0.3cm deep; moderate, 4.0cm long and 0.5cm deep; severe, 4.0cm long and 1cm deep. Patients with a moderate or severe lesion are considered to have “large” defects. Whilst this system was not developed for arthroscopic evaluation, it was easily extrapolated for such use [24]. Flatow *et al*. suggested quantifying the humeral bone loss as a percentage of involvement of the humeral head *i.e*. less than 20%, 20 - 40% and greater than 40%. Lesions >40% are clinically significant [25]. There have been numerous attempts at further classifying HSLs. However, the rarity of these lesions makes it difficult in applying and validating classification systems as well as gaining consensus by majority of surgeons.

## 11 TREATMENTS

### 11.1 Conservative Management

Management of these patients remains controversial and is based on numerous patient factors including activity level and chronicity, anatomical features and surgeon factors. A trial of non-operative therapy is warranted in most patients, including immobilization before the initiation of physical therapy focusing on the dynamic shoulder stabilizers. This normally allows time to further investigate the instability associated pathology and surgical plan. For elderly patients with large HSLs who are high risk for complications following surgery and anaesthesia or low demand individuals, the best option usually is conservative management *via* extensive physiotherapy. Their rehabilitation program must focus on improving deltoid and the rotator cuff muscles function, as well as the scapular stabilizers.

### 11.2 Surgical Management

The aim of surgery is to reduce instability through the least invasive method. Many of the surgical procedures are technically challenging and carry a risk of morbidity. There is also the risk of hardware failure and a reduced range of motion. The least invasive procedures are therefore growing in popularity.

Prior to considering surgical options one must look at many factors. These include the size of the lesion, location, the patient’s age and expectations, whether the lesion involves both the glenoid and humerus or not, bone quality and any previous shoulder stability surgery.

Arthroscopic Bankart repair alone has been effectively used to treat humeral head defects involving 20% of the articular surface. Larger defects involving around 30% of the articular surface have been addressed with humeral rotational osteotomy, osteochondral bone graft and arthroplasty.[26] For those lesions more than 40% of the humeral head, anatomic reconstruction or arthroplasty is indicated depending on patient's age. For patients with bipolar lesions one needs to determine the relative contribution of each lesion and address the most problematic lesion or both as required [26]. A 2016 survey of the American Shoulder and Elbow Surgeons showed consensus between surgeons in their surgical treatment for the above HSLs on initial presentation but less so when revision was required [27].

Currently, the two general ways of achieving stability in patients with HSLs is to either reduce the range of external rotation or fill the humeral head defect. The former includes anterior soft tissue shortening or rotational osteotomy of the humerus. The latter includes bone grafting or soft tissue disposition into the defect or percutaneous transhumeral headplasty. Techniques can also be divided into anatomic or non-anatomic techniques [4].

The Bristow-Latarjet procedure of surgically transferring the coracoid process to the anterior glenoid effectively lengthens the articular arc preventing engagement of the HSL. There is excellent long-term data supporting this procedure [3]. Disadvantages of this technique include the loss of external rotation, coracoid graft osteolysis and glenohumeral arthritis. The procedure is technically challenging and produces a non-anatomical repair and a complicated revision surgery should it fail. Furthermore, the data is from a non-homogenous population as surgeons have different operative thresholds with respect to bone loss. A recent study also observed the direct link between compensation claims with ongoing instability in patients who had undergone a previous Laterjet procedure [28].

### 11.3 Rotational Humeral Osteoplasty

In 1984, Weber *et al* described a rotational osteotomy in the proximal humeral shaft. This increases the retroversion of the proximal humerus redirecting any defect more posteriorly preventing engagement [29]. Rotational osteotomies are associated with a 5.7% re-dislocation rate with 90% of patients having a good to excellent result. 59% required second surgery for removal plates after 1-2 years [29].

### 11.4 Capsular Shift

In this technique the glenohumeral joint capsule is surgically tightened through either open or arthroscopic approach. The tight capsule stabilizes the joint by limiting the external rotation and anterior translation [30]. Capsular plication techniques in conjunction with Bankart repair are among the most commonly performed procedures for anterior shoulder stabilisation. However, it doesn’t provide an anatomic solution. The engagement of the humeral head is avoided. The HSL may still rotate to an intraarticular location but will remain stable through a functional range of shoulder motion [10]. A recent retrospective study found a recurrence rate of 13.2% after arthroscopic Bankart repair and capsular shift [31]. The estimated probability of recurrent instability within the first two years after arthroscopic Bankart repair and capsular shift showed that in patients with engaging HSLs and glenoid bone loss of 0-5% was between 12-38% (decreasing with age) [31]. The risk of recurrence was independently predicted by the patient’s age at surgery, the severity of glenoid bone loss and the presence of an engaging HSL [31]. This is again similar to what other studies have found with contact sports and hyperlaxity. Loss of more than 25% of the glenoid bone was also significantly associated with recurrence [31]. Open capsular repair showed similarly low recurrence rates but with a restricted external rotation and risk of developing secondary osteoarthritis [23].

### 11.5 Remplissage

In 1972, Connolly described this open procedure, which involves transfer of the infraspinatus tendon with a portion of the greater tuberosity into the humeral head defect. This filling of humeral head defect converts them into extra-articular lesions [32]. Wolf and Pollack were the first authors to use the phrase “remplissage” (French for “filling”) [33]. This involves arthroscopic posterior capsulodesis and infraspinatus tenodesis by applying sutures in the muscle belly to fill large Hill-Sachs lesions. These structures then provide a mechanical block to instability [33]. This technique was shown to provide effective treatment for HSL in combination with less than 25% glenoid deficiency [34]. In 2009, Koo *et al* modified this technique further using the double-pulley suture technique. They tied two anchor sutures over the infraspinatus tendon rather than the muscle, thereby obtaining a more physiologic and mechanically sound construct, specifically by providing a large footprint of fixation. This eliminates the risk of muscle necrosis due to strangulation by sutures [35]. Remplissage is successfully performed with Bankart repair in patients with moderate to large HSLs associated with glenoid defects of less than 20-25% [36-38]. Many other studies have analyzed the outcome of the remplissage procedure on shoulder stability and range of motion. They concluded that remplissage prevents engagement and enhances stability in lesions as large as 45% defects, but with some loss of shoulder movement [36-38]. Other studies have proved the loss of external or internal rotations to be statistically insignificant when compared to pre-operative range of motion or with Bankart only procedures [39-41]. They describe 68-80% of patients returning to the same level of sports, including those involving overhead activities [39, 42, 43]. A recent case series looking at large engaging HSLs treated with remplissage showed 65.5% of patients returning to throwing sports complained of a decreased range of movement but overall there was a redislocation rate of 11.8% at 5 years [44]. This same study showed a return to full sports at 7 months in 95.5% of patients [44]. More long term studies are required to assess the development of osteoarthritis post-procedure within these patients.

### 11.6 Humeral Head Augmentation

Osteoarticular reconstruction of the humeral head is performed by filling in the humeral head defect with osteochondral allograft. This restores the humeral head anatomy preventing the osseous engagement on the anterior glenoid rim. Multiple surgical approaches have been employed: arthroscopic, anterior deltopectoral only, combined arthroscopic and posterior. There are few published clinical reports of this strategy in HSLs with or without glenoid bone injury. The advantages of this technique over others include restoration of the articular surface and a mechanically stable joint without significantly altering the joint kinematics. Furthermore, future prosthetic reconstruction may be less challenging and more of a suitable option than if a non-anatomical reconstruction was used.

The challenges associated with this approach, however, include an extended deltopectoral approach requiring a subscapularis tenotomy and capsulotomy. In addition, there is risk of cyst formation, graft resorption, nonunion and hardware failure [45]. Miniaci *et al* described a reconstruction of the humeral head using size-matched osteoarticular allograft [46]. At 2 years follow up, there were no recurrent instability and patients had returned to near normal activity. The excessive rotation of the humerus and retraction of local soft tissues required intra-operatively may increase the risk of avascular necrosis and therefore also cause further morbidity. Two out of 18 patients required screw removal for undesired symptoms [46]. Chapovsky and Kelly reported a single case of arthroscopic allograft mosaicplasty of the humeral head in a 16 year old male with a large HSL whose previous arthroscopic anterior repair had failed [47]. Three 5mm plugs where placed along the HSL. He remained asymptomatic post-operatively and returned to basketball at 1 year post operation [47]. Whilst this technique is less invasive it would be limited by the size of the HSL. Use of multiple plugs would limit the biomechanical stability particularly to shear force and there would remain defects between the plugs.

Kropf and Sekiya described another technique of anterior arthroscopic osteoarticular allograft reconstruction of the humeral head [48]. They proposed performing arthroscopic Bankart repair and measuring the humeral head defect. It would be at this stage the surgeon could either stage the procedure or proceed there and then to reconstruct the humeral head. In a staged procedure the patient can return 6 weeks post initial Bankart repair for allografting [48]. Through a posterolateral deltoid and infraspinatus split, the plug is placed in a prepared socket and tapped flush into position. They presented a single case where this staged technique was performed in an active 19 year old enlisted US Navy seaman, with excellent short-term results with the patient returning to active duty by 1 year [48]. The advantage in the staged technique is the limited posterior approach preventing excessive anterior structure sacrifice and excessive humeral head rotation reducing the risk of vascular compromise [48]. As shown in other studies discussed, early intervention in the appropriate population is encouraged to prevent arthritis and maintain function. Further biomechanical studies are underway looking into which size of defects should be treated this way [48].

### 11.7 Humeral Head Osteoplasty/Dissimpaction

Kazel and colleagues described a technique termed “humeroplasty” to either decrease or correct the size of a large HSL in a cadeveric model [49]. It involved disimpaction of the HSL and elevating and supporting the remaining bone with bone graft. Additionally, Re *et al* and Mehta demonstrated how to reduce the HSL lesions through the humeral head with tamps using a deltopectoral approach [50, 51]. Mehta was able to reduce a chronic HSL with good early results [51]. Sekiya *et al* equally used the same technique through an anterior cortical window [52]. This cadeveric work concluded that complete correction was not possible and one could only convert large defects into smaller defects [52]. Another cadaveric study, created HSLs on 18 cadaveric shoulder models and then performed balloon humeroplasty [52]. The average pre-reduction HSL volume was 1515.5mm^3^ and average post-reduction lesion residual volume was 31mm^3^ demonstrating a 99.3% reduction of the original humeral head volume [53]. The presumed advantages of percutaneous technique included it being less invasive compared to the techniques previously described, restoring humeral head concavity and articular arc length without altering the anatomy to prevent engagement of the humeral defect by either rotating the humerus or over constraining the soft tissues affecting rotation and it does not rely on healing of bulk osteoarticular allograft or transposed infraspinatus tendon. Its potential limitations are that it may not be suitable for sufficiently large humeral head defects or for those patients with significant osteopenia where subchondral support is lacking [52, 54]. Other potential complications include further damaging the articular surface, fracture, axillary nerve injury and even compartment syndrome due to the high balloon pressures [52, 54].

## 12 ARTHROPLASTY

Defects greater than 40% of the humeral head can lead to recurrent or permanent dislocation so one must consider prosthetic replacement as an alternative for reconstruction of the proximal humerus. There have only been a few case reports of resurfacing of the humeral head and either hemiarthroplasty or total shoulder arthroplasty in the setting of HSLs or glenoid defects in association with dislocation [55-58]. Hemiarthroplasty or TSA is best suited to older patients with preexisting glenohumeral osteoarthritis and osteopenic bone, who will not benefit from reconstruction and instead may achieve greater postoperative motion and pain control with a replacement procedure.

A case series of 11 patients with fixed anterior shoulder dislocation were treated with either hemiarthroplasty (6) or TSA (5) [55]. Four of the hemiarthroplasty group had a combined anterior glenoid reconstruction. Eight patients reported excellent or good outcomes with the remaining three reporting it to be fair. During the follow up of four years, seven complications in five shoulders were observed. Due to glenoid loosening, four cases had recurrent anterior dislocations. Removal of metalwork and removal of the glenoid component were performed in two separate cases [55]. Whilst a reliable treatment for shoulder pain, limited functional results can be expected from arthroplasty. For younger patients with greater than 40% HSL there is an option of humeral head resurfacing. Raiss *et al* reported 10 patients with traumatic fixed anterior glenohumeral dislocation treated with the cementless humeral surface replacement arthroplasty (CHSRA) for a mean follow-up of two years [59]. Good clinical outcomes with a moderate complication rate was observed. No signs of implant loosening were seen although sufficient bone stock is required for stability. Bone defects of more than 45% of the humeral surface is suggested to be the limit for this procedure and when one should consider a hemiarthroplasty. The authors suggested that younger patients *i.e.* under 50 years would be more suited to humeral head preserving surgery techniques [59]. Focal resurfacing of the defect with an implant is another option for younger patients. In a case series of two patients, Grondin and Leith used a HemiCAP metal implant to cover large HSLs. However, they had to perform a Laterjet procedure to help stability as both patients had associative bony Bankart lesions. They reported good functional outcomes [60]. Benefits included the preservation of bone stock, which makes future revisions easier and restoration of the normal articular surface without the technical difficulty of precisely matching osteochondral grafts to the articular curvature. More long term follow up results are needed as the long-term survivorship rates are not known.

## 13 NEW TECHNIQUES

Kyphoplasty, more often seen in reducing vertebral compression fractures, has recently been used to reduce HSLs in cadaveric studies almost achieving an anatomical correction [61]. This could be a better option for HSLs reducing surrounding tissue damage that can occur with other minimally invasive techniques. This new technique might behave differently when performed on normal individuals [61].

## 14 REVERSE HILL-SACHS LESION

Posterior shoulder instability is uncommon, since it accounts for less than 5% of all episodes of instability. It is associated with bony or ligamentous disruption [62, 63]. Approximately 30% of posterior dislocations lead to an anterior impression fracture of the anterosuperomedial humeral head, termed the reverse HSL [62, 63]. In these patients the damage is a lot more extensive than seen in anterior dislocations. These dislocations are usually secondary to high-velocity trauma, falls, epileptic seizures and electrocution. In patients with epilepsy, the relatively stronger internal rotator muscles contract and overpower the weaker external rotators [26]. Patients may present with a locked irreducible posterior dislocation in which the humeral head engages the glenoid rim. The patient therefore holds their arm and shoulder in internal rotation and there is a marked loss of external rotation [64, 65]. AP and lateral radiographs classically show a “light bulb” sign and possibly the reverse HSL. Imaging such as CT or MRI is often crucial in detecting associative injuries [64, 65]. After reduction, if the remaining lesion is less than 20% then the shoulder joint can be initially managed non-operatively either by immobilisation in neutral or preventing external rotation for around four weeks [62]. Elderly and low demand patients with chronic dislocation may cope well with minimal pain and just enough shoulder mobility to perform activities of daily living [26]. As with all dislocations, recurrent instability necessitates surgical stabilization. Repairing just the capsule and labrum is not enough, especially if the shoulder is locked posteriorly [66]. Reoperation rates are as high as 20% in many series [67]. Lesions up to 50% of the articular circumference have been treated with anatomical reconstructive techniques whilst lesions larger than this may require arthroplasty [68]. For small impression fractures traditional treatment involves detachment and transposition of the subscapularis tendon (Neer’s modification) and or the lesser tuberosity (McLaughlin procedure) with transfer into the humeral defect using an open anterior approach. Successful stability has been achieved with these procedures [69]. They are less successful for lesions between 20- 40% [68-70]. The complications of Neer’s procedure include altering the anatomy in such a way that can lead to subscapularis dysfunction and if required, a more complex arthroplasty surgery [26]. In 2006, Krackhardt *et al* described an arthroscopic subscapularis tendon transfer to the defect using suture anchors. This treated the defect and prevented extension during internal rotation avoiding redislocation. Twelve of these had been reported without any major complications [71]. Rotational osteotomy has similar results for posterior dislocation as for anterior. It allows for improved shoulder range of motion, but does limit external rotation. Concerns due to its technical difficulty and increased risk of devascularization have meant it is rarely utilised [72]. The posterior bone block is a good treatment option for posterior dislocation and has good short-term results with low recurrent dislocation rates despite the increased risk of glenohumeral arthritis [73, 74]. Osteochondral bone grafting can also be considered for patients with medium to large reverse HSLs *i.e.* 20-40%. Femoral head allograft to fill large reverse HSLs have showed good functional results with no instability. Diklic *et al* presented thirteen cases with chronic unreduced posterior dislocations of the shoulder with associative humeral head defects *i.e*. 25-50% of the articular surface. They reconstructed the defect with femoral head allograft. Patients reported good pain relief, stability and function at a mean follow up of four and a half years. The drawback with this technique is that it requires good bone quality of the humeral head and no glenohumeral osteoarthritis [75]. In a more recent study, two patients had their impression defect (less than 35%) elevated and then filled with allomatrix bone graft putty. This construct was then stabilized with raft screws and so it can only be done to none fragmented lesions. After initial bracing for four weeks to prevent internal rotation they were then allowed full active range of movement. At two years functional results were excellent. The authors proposed that this technique can be used in patients with medium sized (20% to 40%) reverse HSLs [76]. In a recent study, following posterior shoulder dislocation (seven to eight weeks), six men underwent allogenic grafting with humeral head contouring of medium sized (40%) reverse HSLs [77]. By four months, all patients had returned to their occupation. Three patients had an excellent clinical outcome matching radiological observation. One other was 8 years post-surgery who developed osteoarthritis and went on to have arthroplasty surgery. The final two suffered collapse of the graft requiring arthroplasty [77].

## CONCLUSION

Arthroplasty should be reserved for dislocations associated with large humeral head defects, severely damaged humeral heads or osteoarthritis of the humeral head. Gavriilidis *et al* performed arthroplasties in patients who were symptomatic with greater than 45% damage to the humeral head articular surface. Ten patients underwent hemiarthroplasties and two had TSAs. At a mean follow up of 37.4 months, function and patient satisfaction had improved with no recurrence of instability. One patient required revision at 36 months with polyethylene insert exchange. Two patients developed migration of the humeral head but neither required revision [64].

## LIST OF ABBREVIATIONS

ALPSA: Anterior Labroligamentous Periosteal Sleeve Avulsion
CT: Computerised Tomography
CTA: Computerised Tomography Arthrography
HSL: Hill-Sachs Lesion
MRI: Magnetic Resonance Imaging
US: Ultrasound
